# Capturing red squirrels (*Sciurus vulgaris*) on camera: A cost‐effective approach for monitoring relative abundance and habitat preference

**DOI:** 10.1002/ece3.10536

**Published:** 2023-10-03

**Authors:** Graeme Shannon, Simon Valle, Craig M. Shuttleworth

**Affiliations:** ^1^ School of Natural Sciences Bangor University Bangor UK; ^2^ IUCN Species Survival Commission Conservation Planning Specialist Group St. Paul Minnesota USA

**Keywords:** conservation, forestry, population survey, small mammal ecology, study design, wildlife management

## Abstract

Effective methods for monitoring animal populations are crucial for species conservation and habitat management. Motion‐activated cameras provide an affordable method for passively surveying animal presence across the landscape but have mainly been used for studying large‐bodied mammals. This paper explores the relative abundance and habitat preferences of red squirrels (*Sciurus vulgaris*) in coniferous forests using cameras and live trapping. The study was conducted in two forests (Newborough and Pentraeth) on Anglesey, North Wales, with a total of 50 sampling locations across four habitat categories. Detailed woodland structure and composition data were gathered around each sampling location. We found a strong positive correlation between the number of individual red squirrels live trapped over 10 days with the number of camera images of squirrels recorded during a previous 5‐day period. The time interval between camera deployment and the first recorded image of a red squirrel showed a significant negative correlation with the number of individuals live trapped. Red squirrel relative abundance was negatively related to forest canopy openness, while the presence of Scots pine and increased tree species diversity were positively associated with the relative abundance of squirrels. There was also a strong site difference with lower relative abundance at Newborough compared with Pentraeth, which likely reflects the heavy thinning of mature forest at Newborough reducing tree crown connectivity. The results show that remotely activated cameras are an effective method for monitoring red squirrel populations across varying animal densities. The cameras also provided crucial information on red squirrel habitat preferences that can aid in woodland management and conservation efforts. Cameras have great potential to collect data on the population status of other small mammals, but it is essential that these methods are validated on a species‐by‐species basis.

## Graphical abstract

Monitoring animal populations is crucial for effective conservation. However, resources are often limited, which necessitates the development of rapid and reliable survey methods. We demonstrate how motion‐activated cameras can be used to measure the relative abundance and habitat preference of red squirrels across a range of woodland habitats.

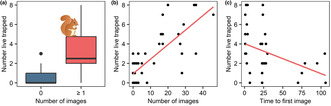

## 1 INTRODUCTION

Estimates of population size and trends in local abundance are essential for the development of coherent plans to conserve and manage wildlife (Jones et al., 2013; Newson et al., 2008), and they form the basis of the IUCN Red List assessments (IUCN, 2022). Effective monitoring enables conservation scientists and practitioners to gain detailed biological insights on species, detect demographic changes and inform policy and direct management interventions (Jones et al., 2013). Ideally, abundance estimates for species of conservation interest should derive from high‐quality data collected during standardised surveys that are repeated over time and account for differences in detectability (Upton, 2020; Williams et al., 2002). However, there are many species of conservation concern and the resources available are often limited (Bottrill et al., 2008). Accurate estimates can be labour‐intensive and invasive, particularly in small and medium‐sized mammals where the capture and handling of individuals are often required (Bertolino et al., 2009). Thus, practical, rapid and inexpensive methods to provide reliable indices of animal abundance are much needed (Palmer et al., 2018; Parsons et al., 2021).

The increasing technological sophistication, improved reliability, affordability and passive data collection of camera traps make them an ideal tool for wildlife monitoring and research (Burton et al., 2015). Images can be individually data‐logged and visible species can be readily identified and recorded. Camera traps continually collect data for weeks or months at a time, while the relative ease of deployment and maintenance optimise landscape‐scale data collection (Kays et al., 2011; Steenweg et al., 2017). They have been effectively used to measure the species richness, abundance, occupancy, habitat use and behaviour of elusive large‐bodied mammals (Burton et al., 2015; Caravaggi et al., 2017; Shannon et al., 2014). An increasing number of studies have explored the potential for cameras to collect detailed demographic and behavioural data on small mammals (Glen et al., 2013; Parsons et al., 2021). This approach has the benefit of being less invasive and labour intensive than traditional live‐trapping methods.

Small mammals have a significant impact on ecosystem function, consuming seeds and serving as prey for predators (Lacher et al., 2019), while their presence and abundance make them appropriate ecosystem health indicators (Avenant, 2011; Pearce & Venier, 2005). Small mammals are also a highly diverse group, and many species are threatened by human activities, making them a conservation priority. Despite this, they often receive less research attention compared to larger‐bodied taxa (Verde Arregoitia, 2016). The red squirrel (*Sciurus vulgaris*) is a small rodent native to Europe, weighing between 270 and 320 g (Lurz & Lloyd, 2000; Wauters & Dhondt, 1989). It is well adapted to arboreal forest life and consumes a diet dominated by tree seeds (Krauze‐Gryz & Gryz, 2015; Moller, 1983). Changes in squirrel densities are a clear indication of annual fluctuations in tree seed abundance (Lurz et al., 1995; Wauters et al., 2008). Additionally, higher population densities occur in habitats with a lower annual variation of seed availability, while body mass, winter survival and reproductive success are all positively correlated with tree seed production (Wauters & Dhondt, 1989; Wauters & Lens, 1995).

Red squirrel numbers in the United Kingdom (UK) declined dramatically over the last century due to habitat loss and the introduction of the eastern grey squirrel (*Sciurus carolinensis*) from North America in the late 1800s. The grey squirrel can outcompete red squirrels for food resulting in elevated stress and reduced fecundity (Gurnell et al., 2004; Santicchia et al., 2018). Moreover, in the UK and Ireland, the grey squirrel is an asymptomatic reservoir host of squirrelpox virus (Bruemmer et al., 2010), an infection‐causing disease in red squirrels with population mortality rates exceeding 80% (Chantrey et al., 2014). Applied national conservation efforts have focused on limiting the threats of competition (Wauters et al., 2000; Wauters & Gurnell, 1999), stress (Santicchia et al., 2018) and disease transmission (Chantrey et al., 2014; McInnes et al., 2013; Rushton et al., 2006) from grey squirrels. However, there is a lack of a ‘cost‐effective monitoring tool’ to assess the response of red squirrel populations to sympatric grey squirrel control or woodland habitat management (Wales Squirrel Forum, 2018).

Camera trap detection rate (number of camera images of target species/unit time) has been proposed as a metric of relative abundance in studies where individual animals cannot be identified (i.e. unmarked). The first evidence of the utility of this method was the linear relationship generated between local densities and rates of camera trap images for tigers (*Panthera tigris*) across Asia (Carbone et al., 2001). Despite initial scepticism around the calibration of the method (Jennelle et al., 2002), this approach has since been tested and used across a range of mammalian taxa including carnivores (Linkie et al., 2006), primates (Garriga et al., 2019) and savanna ungulates (Palmer et al., 2018; Rovero & Marshall, 2009). Refinements to this methodology now incorporate metrics of detection and movement behaviour to improve the accuracy of density estimates for these large‐bodied species (Gilbert et al., 2021; Rowcliffe et al., 2008). Initial studies have highlighted the potential of determining the relative abundance of smaller‐bodied mammals using camera traps, but this is highly dependent on study design and target species biology (Parsons et al., 2021; Weerakoon et al., 2014). As such, comparing the performance of camera traps against established methods for determining relative abundance is crucial for evaluating their contribution to conservation monitoring and research.

To the best of our knowledge, there are no published studies on the efficacy of camera traps to estimate local red squirrel relative abundance, although Villette et al. (2017) did use cameras to explore the densities of the North American pine squirrel (*Tamiasciurus hudsonicus*). Currently, mark‐recapture studies have provided the most accurate means of determining local red squirrel populations (Kenward & Holm, 1993; Lurz, 1995; Villette et al., 2017; Wauters et al., 2008). However, red squirrels are legally protected in the UK and a licence is required to live capture, handle and mark the species. Live trapping is also labour‐intensive and logistical considerations mean that the spatial scale of any operation is limited. Distance sampling along transects offers a non‐invasive survey method that does not require a licence (Chantrey et al., 2014), but it has limited reliability in woodland habitats with low squirrel densities or in conifer plantations, due to poor detectability (Gurnell et al., 2002, 2004). Hair‐tube monitoring has proved a more effective non‐invasive sampling approach (Bertolino et al., 2009), which can also be used for collecting DNA (O'Meara et al., 2018). However, it requires technical skill and the use of magnification equipment to accurately identify red squirrel hairs. If grey squirrels are sympatric, then hairs need to be stained and cross‐sectioned to confirm species identification; a process that is costly and time‐consuming (Foran et al., 1997; Trapp & Flaherty, 2017).

Our study aimed to explore whether camera traps could provide an inexpensive and standardised method for determining local relative abundance of red squirrels in woodland habitats. We had two objectives: (1) investigate whether camera variables (time to first image capture and cumulative number of red squirrel images) provided robust index of local relative abundance and (2) quantify how live‐trap‐based estimates of red squirrel relative abundance relate to habitat characteristics (stand age, canopy openness, tree diameter at breast height and tree species present).

## 2 METHODS

### 2.1 Study sites

**FIGURE 1:**
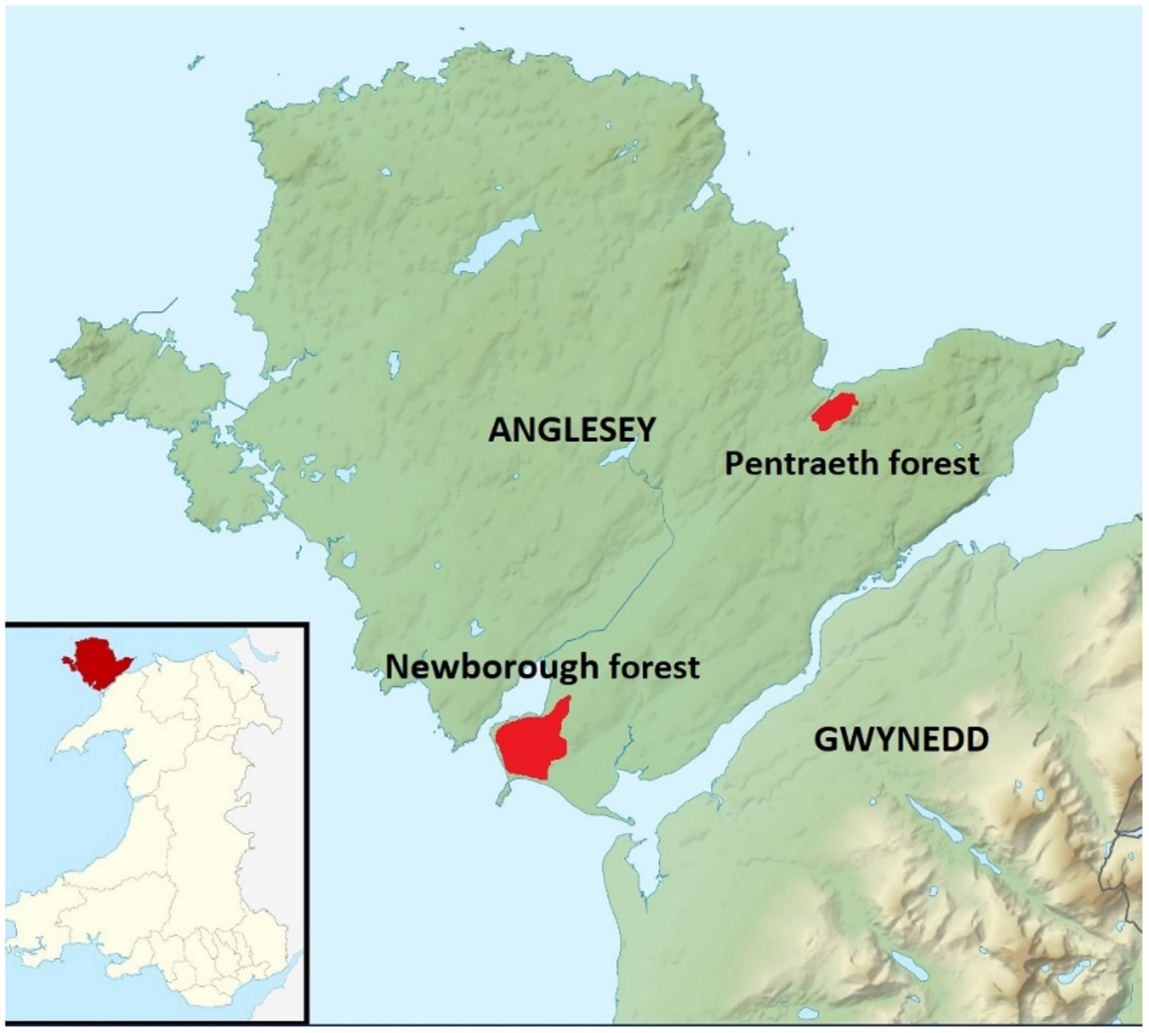
The location of study forests on the island of Anglesey (licenced under the Creative Commons Attribution‐Share Alike 3.0 Unported licence https://creativecommons.org/licenses/by‐sa/3.0/deed.en.Attribution: Contains Ordnance Survey data © Crown copyright and database right).

The study was undertaken in the commercial coniferous forests of Newborough and Pentraeth on the island of Anglesey, North Wales (Figure 1) from March to May 2022. Both forests are managed by Natural Resources Wales, the government‐sponsored body that manages and protects the natural assets of Wales. They were established in the late 1950s–early 1960s and contain first‐ and second‐rotation non‐native coniferous stands with small areas of broadleaved woodland. Newborough comprises 700 ha of coastal habitat that is dominated by mature Corsican pine (*Pinus nigra*), which has been heavily thinned in places. Natural regeneration comprises mainly broadleaved species including the common birch (*Betula* spp.) and willow (*Salix* spp.), while Sycamore (*Platanus orientalis*), hawthorn (*Crataegus monogyna*) and cherry (*Prunus avium*) also occur but are much less common. Coniferous regeneration is limited to areas of Monterey pine (*Pinus radiata*) and small areas of lodgepole pine (*Pinus contorta)*. Young conifer and older plantations are relatively uncommon and mostly dominated by Corsican pine. In contrast, Pentraeth is 244 ha in size and is a mixture of Sitka spruce (*Picea sitchensis*), European larch (*Larix decidua*), Lodgepole pine and Scots pine. Small but significant areas of mixed deciduous woodland are dominated by beech (*Fagus sylvatica*), sessile oak (*Quercus petrea*) and wych elm (*Ulma glabra*). Young and 20–40‐year‐old plantations are typically dominated by Scots pine, larch and Sitka spruce, sometimes in monoculture. There are also extensive areas of birch‐dominated regeneration. In contrast to the selective thinning at Newborough, coniferous stands in Pentreath are typically line thinned.

Grey squirrels were abundant on Anglesey in the late 1990s, and Pentraeth forest was the last woodland containing red squirrels (Shuttleworth, 2003). Subsequently, intensive grey squirrel control facilitated local red squirrel recovery (Schuchert et al., 2014), and red squirrels were reintroduced to Newborough forest in 2004 (Shuttleworth et al., 2016). Anglesey no longer has a grey squirrel population, which means that woodland management is not limited by the need to avoid planting tree species (e.g. oak, hazel), which may benefit grey squirrels and exacerbate their competitive advantage (Lurz et al., 2004). However, pine species – in particular Corsican pine – are vulnerable to partial defoliation caused by red band needle blight (*Dothistroma septosporum*) (Brown et al., 2003). Consequently, continuous cover selective thinning of affected Corsican pine stands has occurred to open the forest canopy, maximise air movement and reduce fungal infection (Bowne, 2010).

### 2.2 Survey design

Natural Resources Wales provided data on area, species composition and planting date for each spatial forest management unit for both forests. Sampling locations were selected to be proportionally representative of four key habitat types across both forests (Table 1). This was due to the limited size of the study sites, their close proximity and relatively high degree of patchiness. Where units had been planted with a mix of species (particularly in Pentraeth), we categorised them according to the dominant species, that is, ≥50% of the area planted. Ground truthing revealed that some stands had been clear‐felled and these areas were excluded from the study.

**TABLE 1:** Extent of the woodland habitat types across the two study sites.

Category	Area (km^2^)	% of total
Broadleaved	0.651	7.1
Young Conifer (1–19 years of age)	0.452	4.8
Conifer 20–40 years of age	1.086	11.6
Conifers 40+ years of age	7.142	76.5

Fifty sampling locations were selected across the four habitat type categories that would be as central to the forest unit as possible and no less than 250 m away from any other given sampling location (to maximise independence). Given that both Newborough and Pentraeth are characterised by a heterogeneous mix of different habitat types, which vary greatly in size, it was not possible to locate the sampling locations at random without compromising their independence from each other and adjacent habitats. An initial scoping site visit was conducted to establish their accessibility. Where terrain or site conditions prevented access, or where a point was close to a track or public footpath it was moved to the nearest suitable location. This selection protocol resulted in 20 sampling locations in Pentraeth and 30 in Newborough.

The four habitat types differed in extent (Table 1) and therefore the number of sample plots per habitat type varied, that is, 11 in Broadleaved, 8 in Young Conifer, 10 in Conifer 20–40 years of age, and 21 in Conifers 40+ years of age. At each sampling location, we collected data on vegetation structure and characteristics (see Section 2.3). After having recorded habitat data at each sampling location, we scattered the ground with 1500 mL of black sunflower seed. Baiting was done within 3 h of dawn. Five days later a squirrel feeder filled with sunflower seed was erected (again within 3 hours of dawn), and a Browning Recon‐force remotely activated camera with a 64GB card was set up using a standard uniform series of settings. (see Section 2.4).

Cameras were in situ for five full days. In the early morning of the sixth day, the camera and feeder were removed, and a live‐capture trap was set at each survey point. Live trapping was conducted for five cumulative days based on typical methodologies (see Section 2.5 & Gill et al., 2019 for details of live‐capture approaches). A second 5‐day live trapping session was carried out between 23 and 45 days later so that for each survey point there was a cumulative 10 days of live trapping data collected. The second session reflected a likelihood that some animals present at the location may not have been caught during the initial 5‐day live trapping period.

### 2.3 Habitat characteristics

Five replicate sets of habitat data were recorded at each sampling location. The first set was recorded at a central point and the remaining four at a 25‐pace distance in the four cardinal directions. All measures were then averaged across sample plots to give a single value per location. At each point, we assessed canopy openness using a type‐A spherical densitometer (Forestry Supplies; Jennings et al., 1999; Lemmon, 1956, 1957). We collected four measurements per replicate, the recorder turning their body 90° clockwise after taking each reading. An average of the four values was calculated and converted into a ‘percentage of canopy openness’ (hereafter just ‘canopy openness’).

A measuring tape was used to determine the trees contributing to the canopy within a 5 m radius of each sampling point. The tree species and diameter at breast height (DBH) in cm were recorded for each tree. Thus, at each point, a cumulative total of 20 canopy measurements and five sets of DBH were obtained as well as data on tree species composition. Tree species were later categorised as being of ‘low’, ‘medium’ or ‘high’ value for red squirrels (as in Gurnell et al., 2002). ‘Low’ encompassed willow, alder (*Alnus glutinosa*), birch, rowan (*Sorbus aucuparia*), ash (*Fraxinus excelsior*), horse chestnut (*Aesculus hippocastanum*), poplar (*Populus* sp.), hemlock (*Tsuga heterophylla*), holly (*Ilex aquifolium*), silver fir (*Abies alba*). ‘Medium’ included oak (*Quercus* sp.), sycamore, Sitka spruce, Weymouth pine (*Pinus strobus*) and Douglas fir (*Pseudotsuga menziesii*). ‘High’ encompassed Lodgepole pine, Scots pine, Norway spruce (*Picea abies*), hazel (*Corylus avellana*), sweet chestnut (*Castanea sativa*), beech (*Fagus sylvatica*) and cherry. The number of woody stems within a 5 m radius was counted at each of the five points and then averaged for each sampling location to provide a measure of forest regeneration. The total number of tree species per point was also calculated. The presence or absence of Corsican pine, lodgepole pine, Scots pine and larch were specifically noted for each sampling point.

### 2.4 Camera settings

We used Browning Recon Force 4K (BTC 74K) motion‐activated cameras with infrared flash. The cameras were programmed to take three images per detection at a high (16MP) resolution setting. Each detection was followed by a delay of 30 s before any further images would be recorded. We used a ‘normal’ sensitivity setting and a ‘normal’ trigger range. Each camera was positioned so that a wooden squirrel feeder was central in the field of view. The feeder was fixed to a tree trunk at a height of 1.2–1.5 m. Cameras were attached to a tree 1.2–2.4 m from the feeder. After 5 days of operation, the camera data were uploaded to the open‐source software digikam (www.digikam.org), which enabled the presence/absence of a red squirrel to be tagged in the meta‐data of each image. The metadata was then extracted for analysis in R (R Core Development Team, 2022) using the *camtrapR* package (Niedballa et al., 2016). Two key metrics were extracted from each camera: (1) the total number of red squirrel images over the 5‐day period, which included a minimum of 10 minutes between successive sightings to ensure that the data set predominantly focussed on independent foraging events (Parsons et al., 2021; Villette et al., 2017) and (2) the time in hours taken for the first squirrel to visit the feeder.

### 2.5 Live trapping

Live trapping, squirrel handling and marking activities were carried out under Wildlife & Countryside Act (1981 *as amended*) licences (S087309/1‐2 and S091126/1) issued by Natural Resources Wales. We used unmodified commercially available Albi™ single‐catch Mink traps with solid steel doors and treadles. Live traps were baited with sunflower seeds in husks and cleaned with Virkon S™ disinfectant between all live captures. All traps were opened early each morning and inspected twice before being closed until the following day. Live captured red squirrels were handled in a Virkon S™ sterilised wire mesh handling cone. Individuals were sexed, weighed to the nearest 5 g with a 1000 g Pesola™ spring balance, had their hind shin measured to the nearest 0.1 mm and their reproductive state categorised (Kenward & Holm, 1993; Wauters & Lens, 1995).

During their first live capture session, each individual was marked using a unique Passive Integrated Transponder (PiT tag; Peddymark™ 1.4 × 10 mm), which was inserted under the skin at the nape of the neck or dorsal area of the back where the skin was relatively loose. This facilitated individual identification by scanning for a PiT tag using a Peddymark™ ISO PM450 Scanner. Individual red squirrels with a weight greater than 240 g were defined as ‘adult’. We recorded the survey point location for every live capture or recapture during both the first and second trapping periods in each forest. Data from young animals (<240 g) that were live trapped was excluded from further analysis, as they were all considered individuals that had not been weaned during the initial camera deployment. Research approval was granted by the ethics committee at Bangor University.

### 2.6 Data analysis

All statistical analyses were performed in R (R Core Development Team, 2022). A Kruskal–Wallis analysis was carried out to compare the number of individual squirrels live trapped at sites where the cameras detected at least one image of a squirrel (*n* = 35) with those where no images were recorded (*n* = 15). A Pearson's correlation was then used to explore the relationship between two key camera trap metrics – cumulative number of images and the time to the first image after camera deployment – and the number of individual squirrels that were live trapped at each site over a period of 10 days.

The second stage of the analysis employed a Generalised Linear Model (GLM) with a Poisson distribution (*lme4* package; Bates et al., 2015) to explore how habitat characteristics affected the numbers of individual squirrels live trapped at each of the survey points (*response variable*). Nine explanatory variables were included in the analyses: *habitat type* (broadleaf, conifer 40+ years, conifer 20–40 years and conifer <20 years), *site* (Pentraeth or Newborough), *canopy openness*, *number of stems*, *DBH*, *number of tree species* and the *presence/absence of four key tree species*: Scots pine, Corsican pine and larch.

Data exploration confirmed that the response and explanatory variables met the assumptions of the model (i.e. independence of the data, even distribution of the residuals, correct designation of the variance structure and a linear relationship between the response and explanatory variables). However, two of the stand categories (conifer <20 years and conifer 20–40 years) were combined due to the limited sample size preventing model convergence. This led to there being three stand types ‐ broadleaf, conifer <40 years and conifer >40 years. Only DBH and the number of stems were collinear and these explanatory variables were therefore excluded from occuring in the same models.

A total of 54 candidate models were generated from the nine predictor variables, which included a null model, all single‐factor and two‐factor model combinations and 10 three‐factor models. Akaike's Information Criterion adjusted for small sample size (AICc) was used for model selection (Burnham & Anderson, 2002). The *AICcmodavg* package was used to extract AICc scores and model weights for each candidate model (Mazerolle, 2016). Model averaging was conducted across models accounting for ≥0.95 of the AICc weight to extract parameter *β*‐estimates and their 95% confidence intervals (CI). The significance of the results was assessed by whether the 95% CI overlapped zero. Although multicollinearity was not an issue in this study, we also checked that the direction and magnitude of the *β*‐estimates for each explanatory variable were comparable across the top models, given some of the potential limitations of model averaging raised by Cade (2015). Data are provided as part of the supplementary material (Table S1).

## 3 RESULTS

### 3.1 Live trapping summary

A total of 118 individual squirrels were live trapped across the sampling locations during the two 5‐day trapping periods with a range of 0–8 individual squirrels per sampling location (mean ± SD = 2.4 ± 2.4). Squirrels were live trapped at 35 (70%) of the 50 sites, while there were six sites where squirrels were live trapped, but no camera trap images were recorded.

### 3.2 Camera trapping summary

A total of 15,739 images were captured over the 5‐day sampling period across all the sampling locations. 12,286 of these were of squirrels (78%). These images were down‐sampled to achieve independence between foraging events using a minimum of 10 min between successive photos (Parsons et al., 2021; Villette et al., 2017), resulting in a final data set of 474 squirrel detections. Thirty‐four (68%) of the 50 cameras returned at least one image of a squirrel, while five cameras recorded images of squirrels but no animals were ever live trapped. The time to first detection (with a confirmed squirrel image) ranged from 1 to 106 hrs (mean ± SD = 28 ± 31) after the survey period began. We also recorded a single detection of a pine marten (*Martes martes*) of unknown age and sex in a conifer habitat (>40 years of age) at Pentraeth. This was the first confirmed sighting of a pine marten on Anglesey (Figure S1).

### 3.3 Camera trapping as a red squirrel monitoring method

**FIGURE 2:**
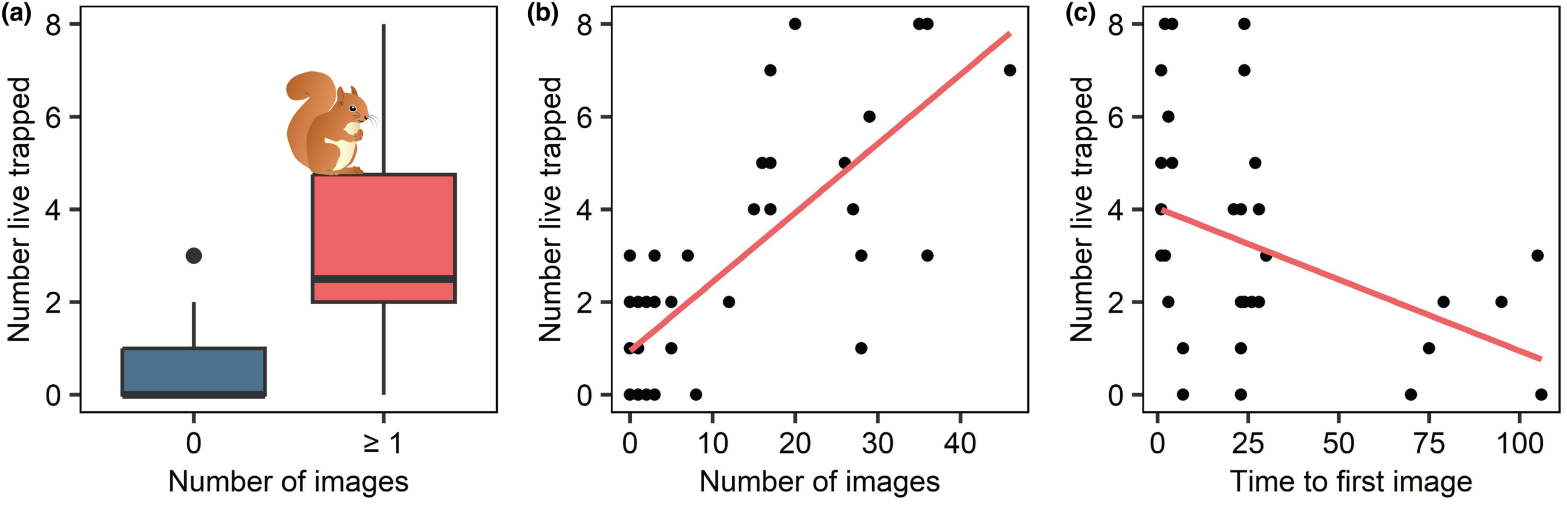
(a) The number of individual squirrels live trapped at sampling locations where the cameras had detected at least one individual compared with cameras that had no detections. (b) Correlation between the number of individual squirrels live trapped and the number of images recorded at each survey camera. (c) Correlation between the number of individual squirrels live trapped and the time to first image at each survey camera in hours.

Significantly more individual squirrels were live trapped at sampling locations where the camera detected at least one image of the target species compared with cameras that did not record a detection (Kruskall–Wallis χ^2^ = 15.187 df = 1, *p* = <.001; Figure 2a). Pearson's correlation revealed a significant positive association between the number of images captured over the 5‐day study period and the number of individual squirrels live trapped (Pearson's: *n* = 50, *r* = .78, *p* = <.001; Figure 2b). Similarly, there was a significant negative correlation between the time to the first image and the number of individual squirrels live trapped (Pearson's: *n* = 35, *r* = −.39, *p* = .02; Figure 2c), demonstrating that cameras at sampling locations with greater numbers of squirrels detected an individual more rapidly. However, the strength of the association was weaker than for the number of images metric.

### 3.4 The impact of forest characteristics on red squirrel habitat use

**FIGURE 3:**
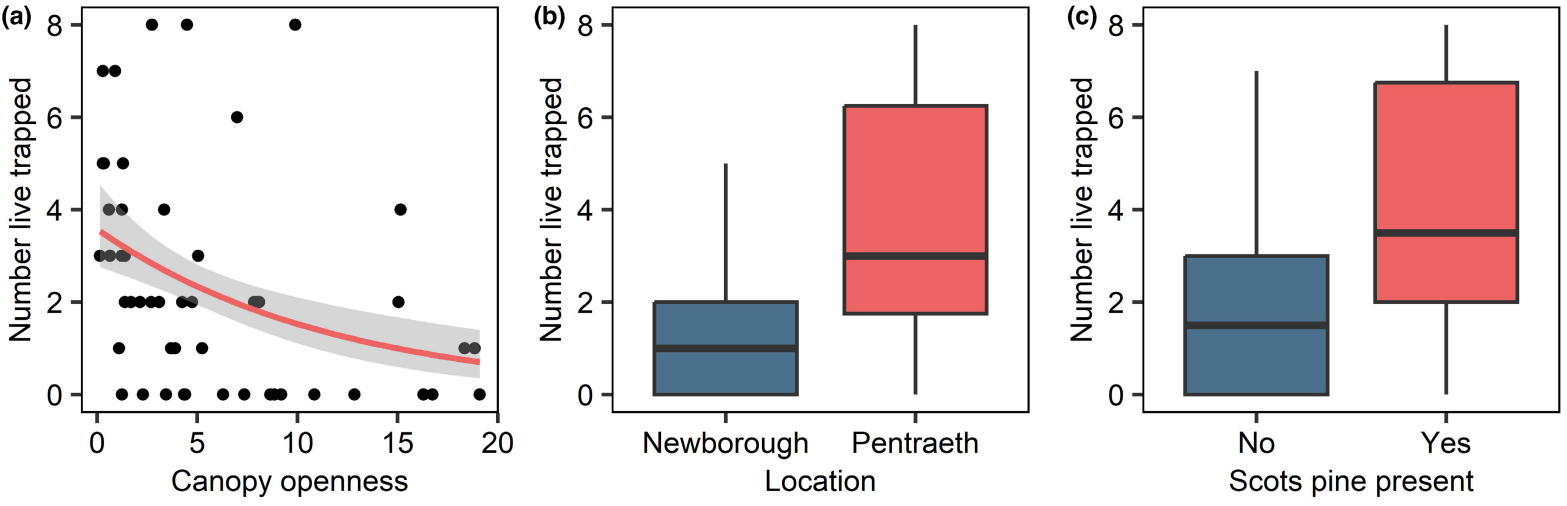
(a) Relationship between the metric of canopy openness and the number of squirrels live trapped at each survey point. (b) The number of squirrels live trapped at the two study sites (Newborough and Pentraeth). (c) The number of squirrels live trapped at survey points which had Scots pine present or absent.

Seven models contributed 95% of the AICc weight for the GLM analysis (Table 2), with the top three models accounting for 78% of the weight. Model averaging revealed that there was a negative association between the number of individuals live trapped and canopy openness (Table 3 and Figure 3a). There was also a site difference in relative abundance with more squirrels live trapped at Pentraeth compared with Newborough (Table 3 and Figure 3b), and a positive relationship between live captures and the number of tree species (Table 3) and the presence of Scots pine (Table 3 and Figure 3c).

**TABLE 2:** Model selection for the GLM analysis exploring the effects of woodland characteristics on red squirrel habitat use.

	*K*	ΔAICc	AICc weight
Canopy openness + Site + Scots pine	4	0.00	0.36
Habitat type + Canopy openness + Scots pine	5	0.60	0.27
Canopy openness + No. tree species + Scots pine	4	1.77	0.15
Canopy openness + Site + No. tree species	6	3.78	0.05
Canopy openness + Scots pine	3	3.94	0.05
Habitat type + Canopy openness + Site	5	4.33	0.04
No. tree species + Site + Scots pine	4	4.94	0.03

*Note*: The models presented in this table accounted for ≥0.95 of the AICc weight.

**TABLE 3:** The observed relationship between the response variable (numbers of individual squirrels live trapped) and the model‐averaged parameters from the top models (*β*‐estimate ±95% CI).

Parameter	*β*‐estimate	95% CI
Habitat conifers <40 years	−0.11	−0.83 to 0.62
Habitat conifers >40 years	0.47	−0.15 to 1.09
**Site (Pentraeth)**	**0.59**	**0.14 to 1.04**
**Canopy openness**	**−0.08**	**−0.13 to −0.02**
DBH	0.01	−0.02 to 0.04
Stems	0.00	−0.02 to 0.01
**No. of tree species**	**0.11**	**0.01 to 0.21**
**Scots pine (presence)**	**0.72**	**0.26 to 1.18**

*Note*: Bold text denotes *β*‐estimates with 95% CI that do not overlap zero.

## 4 DISCUSSION

Our study demonstrated a strong correlation between the number of live captured red squirrels and the number of camera trap images. These results support the use of this approach as an index of relative abundance for a small arboreal mammal. Furthermore, the time to the first image of a red squirrel was negatively correlated with the number of live trapped individuals but this was ultimately a much cruder metric of relative abundance, that is, one that did not account for the intensity of activity across the survey period. Relative indices of abundance, such as those tested here, are a cost‐effective and useful tool when resources and handling skills are limited (e.g. Bertolino et al., 2009; Marsden et al., 2016; Villette et al., 2017). They also greatly reduce disturbance to the target species.

It is important to recognise that there is likely to be greater associated variation with relative abundance estimates from camera traps compared to more rigorous mark‐recapture methods (Parsons et al., 2021). There are also several variables which may further impact the strength of the relationship between camera metrics and live captures across different sampling locations, including the differential use by animals of their home range and individual dispersal (Wauters & Dhondt, 1992). Nevertheless, camera traps are expected to be effective for estimating local relative abundance in habitat patches of varying sizes and with squirrel populations of different densities. Indeed, the method assumes that the sampling effort is representative of the study area size and heterogeneity, that camera trapping locations are independent of each other and the habitat edge and that squirrel activity remains constant over the survey period. At very low densities, the accuracy of the method may be compromised, but it can still provide a relative index of species rarity. It was also notable that we did not record any detections of invasive grey squirrels over the study period, with continued surveillance being central to effective species control (Shuttleworth et al., 2020).

These findings provide further support for the use of cameras in small mammal research and monitoring (Glen et al., 2013; Parsons et al., 2021; Weerakoon et al., 2014). However, it is important to note that the results only apply to red squirrels, and species‐specific calibrations would need to be investigated in other taxa. It is also important to highlight that squirrels were occasionally live trapped at sites where there were no camera detections (and vice versa). Therefore, the absence of detection by cameras should not be taken as evidence of absence unless confirmed by intensive monitoring. Nonetheless, the lack of red squirrels detected by cameras suggests a low relative abundance of the species in that area.

The two metrics we explored differ in their robustness and ease with which they may be used. ‘Number of images’ is a better predictor of local relative abundance but requires a significantly greater investment in data processing, as all the squirrel images need to be individually tagged before being sub‐sampled to ensure that duplicates are removed and there are at least 10 min between successive images. Although ‘time to first image’ is a much easier metric to extract from the data, it is a significantly weaker predictor and should be used with much greater caution, and only if there is no other alternative. Ultimately, the usefulness of both metrics as a proxy for local abundance depends on a rigorous and unbiased sampling strategy that ensures spatial and temporal independence between sampling locations and unique foraging events. (Sutherland, 2006). Our study was conducted in spring, which enabled us to estimate the relative abundance of adults (after over‐winter mortality) during a period that young of the year could be easily distinguished due to their small size. In the autumn months, it is possible that red squirrels will be less attracted to feeders or traps due to the abundance of tree seeds resulting in an underestimate of the relative abundance, but this needs further empirical exploration.

In addition to being an effective and low‐cost monitoring tool for red squirrels, cameras provide insights into their habitat preferences, which can inform conservation interventions. Sympathetic habitat management is crucial for red squirrel conservation (Flaherty et al., 2012; Jones et al., 2016) with reduced tree cover and compromised canopy integrity of woodlands contributing to their decline. Red squirrels show low use of heavily thinned and open canopy plantation forests, as indicated by pinecone exploitation rates (Dylewski et al., 2021; Flaherty et al., 2012). Our research shows that the number of animals trapped was negatively associated with canopy openness. Although red band needle blight may be a factor, it is worth noting that the infection was also present in stands with greater connectivity where squirrels were more frequently found.

Red squirrels preferentially forage near the ‘forest edge’, where low understory cover and high canopy closure characterise the area, and trees usually produce larger cone crops than those growing in the centre of the stand (Dylewski et al., 2021; Turkia et al., 2018). Thinning may increase cone production and reduce competition between trees (Otto et al., 2012), but it can also cause a significant loss in connectivity, potentially resulting in energetic and predation risks for red squirrels while foraging (Flaherty et al., 2012). Although certain thinning regimes may not affect red squirrel space use (de Raad et al., 2021), it is important to avoid assuming that all thinning operations will have minimal consequences for squirrels. Fragmentation of the canopy may force animals to travel along the forest floor if they cannot move between trees, potentially leading to energetic and fitness costs. Additionally, increasing canopy openness may raise predation risk, as lower body conditions and increased movements across the forest floor may make animals vulnerable to predation (Flaherty et al., 2012). It is noteworthy that North American pine squirrels in poor physical condition were more vulnerable to predation (Wirsing et al., 2002). Limiting escape routes for animals attacked by raptors and arboreal predators in the canopy may also increase predation risk.

Another critical consideration is the confirmed presence of pine martens on Anglesey, as these predators are recolonizing Wales and will prey on squirrels (Twining, Montgomery, & Tosh, 2020). Intriguingly, red squirrels have been shown to be significantly less susceptible to pine marten predation compared with predator naïve grey squirrels (Sheehy et al., 2018; Twining, Montgomery, & Tosh, 2020). Evidence suggests that this may be mediated by differences in behaviour, with red squirrels altering their foraging behaviour, vigilance and habitat use in the presence of pine marten scent cues, while grey squirrels did not exhibit anti‐predator behaviours under the same experimental paradigm (Twining, Ian Montgomery, et al., 2020). Future research should attempt to quantify how the presence of pine martens affects the applicability of cameras for assessing red squirrel populations, particularly given their activity around feeding stations may lower squirrel activity and detection probability.

In our study, older coniferous stands generally had higher squirrel relative abundance than younger and broadleaved habitats, but this relationship may be confounded by canopy fragmentation, which is common in stands >40 years old. Low red squirrel presence in broadleaved woodland with intact canopy cover reflects the dominance of birch species, which provide a low‐quality habitat for red squirrels (Jones et al., 2016; Kortland, 2020). Similarly, in >40‐year‐old coniferous stands with heavy thinning, natural regeneration resulted in a dense medium storey of birch, which may improve canopy connectivity but result in a shortage of long‐term food supply when older conifers die or are removed.

Significant differences in squirrel density between Pentraeth and Newborough is likely to result from differences in historical management. Mixed spruce and pine stands at Pentreath were managed with crop line‐thinning before clear‐felling and replanting occurred. In Newborough, continuous forest cover was maintained via selective pine thinning, which involved intensive felling interventions to combat red band needle blight. This, in turn, resulted in a more open canopy. Positive associations between squirrel relative abundance and the presence of Scots pine and tree species diversity likely reflect food resource availability, with Scots pine producing seed crops at an early growth stage. Establishing significant underplanted areas beneath open mature stands using Scots pine in Newborough would not only progressively improve arboreal structure but also could create a valuable future food source for red squirrels in a way that current birch and willow regeneration cannot. Tree species vary annually in their seed production, and a species mix ensures that at least some will produce seed crops each year (Lurz, 1995; Lurz et al., 1995).

## 5 CONCLUSIONS

Our study offers a cost‐effective and straightforward approach to assessing the relative abundance of red squirrels using camera traps. This method can be applied to monitor squirrel numbers at the landscape scale without the need for time‐intensive and costly surveys. The estimate of relative abundance provides a very useful metric for land managers, especially those planning selective forest thinning operations while considering the effects on red squirrels. In addition, our live trapping data has enabled us to explore key woodland characteristics that are driving variation in red squirrel habitat use and activity. The findings demonstrate a strong site difference between Newborough and Pentraeth, which most likely reflects historical management differences, including the heavy thinning of mature stands at Newborough. Older coniferous habitats with greater canopy closure support significantly higher numbers of squirrels. These structural elements of the woodland are also further enhanced by the presence of Scots pine and greater tree diversity, which provide important food sources for the squirrels throughout the year. Our results provide important insights into rapid approaches for surveying red squirrel density as well as guidance on habitat management that can strengthen the long‐term viability of populations across the UK. While our study focused on red squirrels, the use of cameras in monitoring populations of other small mammals may also be promising, though this will require verification on a species‐by‐species basis.

## AUTHOR CONTRIBUTIONS

**Graeme Shannon:** Conceptualization (equal); data curation (equal); formal analysis (lead); funding acquisition (equal); investigation (equal); methodology (supporting); project administration (equal); resources (equal); software (equal); validation (lead); visualization (lead); writing – original draft (equal); writing – review and editing (lead). **Simon Valle:** Conceptualization (equal); data curation (equal); formal analysis (supporting); funding acquisition (equal); investigation (equal); methodology (supporting); project administration (equal); resources (equal); validation (supporting); visualization (equal); writing – original draft (equal); writing – review and editing (equal). **Craig M. Shuttleworth:** Conceptualization (equal); data curation (lead); formal analysis (supporting); funding acquisition (equal); investigation (equal); methodology (lead); project administration (equal); resources (equal); validation (supporting); visualization (supporting); writing – original draft (equal); writing – review and editing (equal).

## Supporting information


Figure S1

Table S1

## Data Availability

The data that supports the findings of this study are available in the supplementary material of this article (Table S1).
