# Supplementary figures and images for: Capturing red squirrels (Sciurus vulgaris) on camera: A cost‐effective approach for monitoring relative abundance and habitat preference
Source: Ecol Evol. 2023 Oct 3;13(10):e10536. doi: 10.1002/ece3.10536 (PMC10546084; doi:10.1002/ece3.10536)

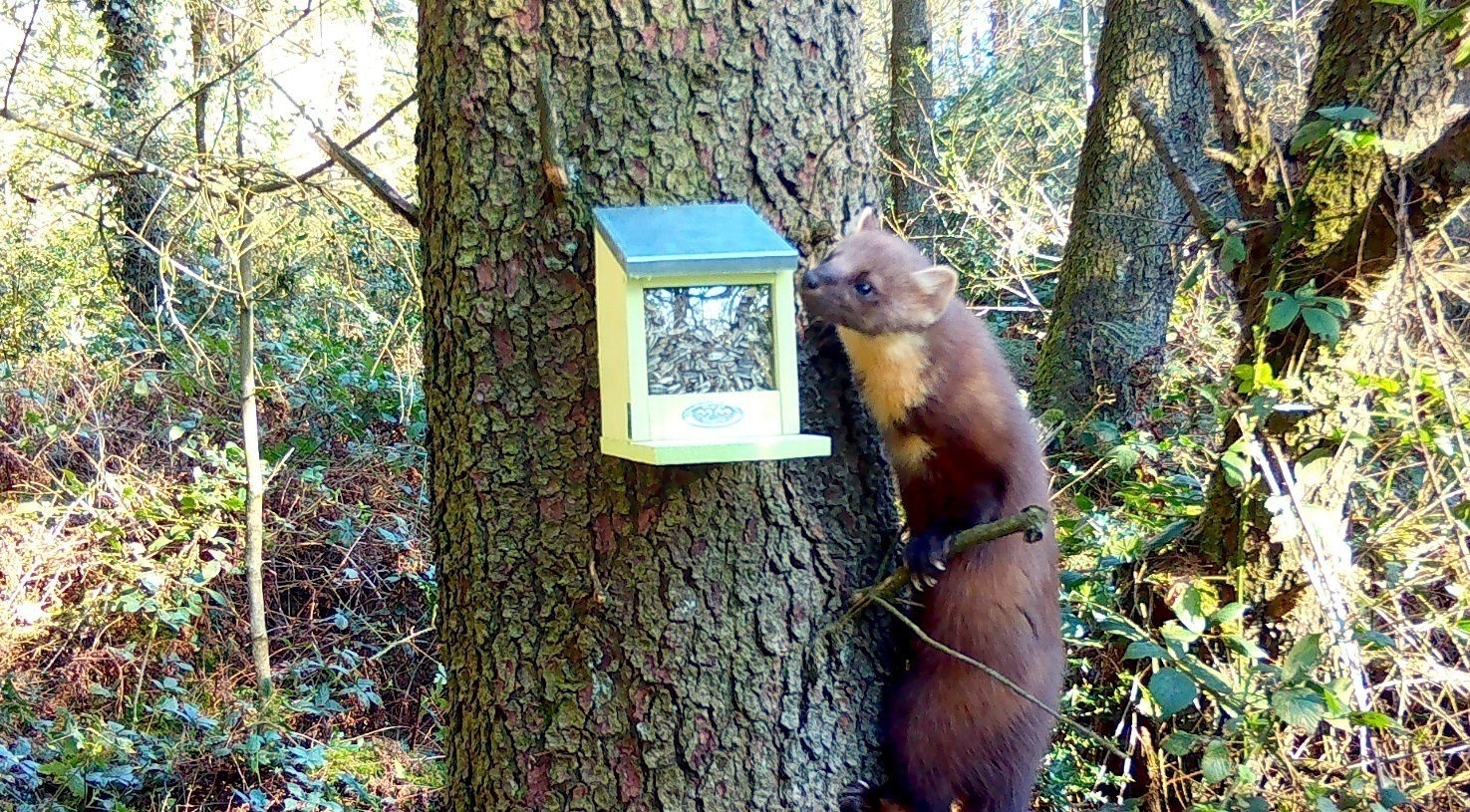


Figure S1. First confirmed sighting of a pine marten on Anglesey.

Supplement: Supplementary file 1 — Figure S1 [file ECE3-13-e10536-s002.docx]
